# Address Delivered before the Graduating Class of the Medical Department of the University of Buffalo at Its Annual Commencement, February 25, 1868

**Published:** 1868-03

**Authors:** J. F. Miner


BUFFALO

### Medical and Surgical Journal.

VOL. VII. | MARCH, 1868. | No. 8.

### Original Communications.

ART. I.—*Address delivered before the Graduating Class of the Medical Department of the University of Buffalo at its Annual Commencement, February* 25, 1868. BY J. F. MINER, M. D.

*Gentlemen:*—Receiving your diploma is very far from ending your labor; it only opens to you another course of education, more pressing in its obligations, and which, in this life is without end. If the diploma has been your object, you are unfitted for the duties of a profession, which imposes upon its members the most sacred obligations to preserve life, and to relieve pain, suffering and sorrow. If your object is to acquire fortune, or honor, or ease, you are as unfit for the profession as the profession is for you. You must look for reward, to the consciousness of being qualified and able to do your duty, and in having done it, even though misjudged and censured, by those you have benefited, and who are incompetent to form any idea of your capabilities, or of the long and anxious labors by which they have been reached. You now belong to a profession which gives you frequent opportunities to practice upon the command, "Do good to those who despitefully use you," and are expected to give up all control of your time, and be constantly prepared for the most momentous and trying emergencies. You must expect to pass anxious hours and sleepless nights from the responsibilities which rest upon you, and the consciousness that the lives of others are dependent upon your skill and judgment. And yet, after all this, you will find very many, from the lowest and most ignorant, to the highest and most learned in other respects, advancing positive opinions in opposition to your own, and recklessly undertaking the care of the sick, and prescribing for their diseases, which is to you such source of mental anxiety and care,

"As fools rush in
Where angels fear to tread."

Such are the contingencies of the new life you have now entered, that you will gladly listen a moment to the reflections and observations of one who has preceded you in the experiences of professional labor, who has met the discouragements and been subjected to the opposing influences to which you will be exposed.

To one educated in the profession of medicine, a very different and much more expanded view is presented, than others, not acquainted with it, are able to gain. He sees in it the various divisions of labor and vast arrangements for it, spread out over the whole civilized world, acting with all the power which can be derived from an aggregation of the highest order of intellect, disciplined and strengthened to the utmost for its work. In every one of the various departments of his profession, the medical student sees a collection of distinguished individuals, whose mental power demands the admiration of all who can appreciate their labors—labors to which nothing short of the greatest intellectual strength is adequate, studying man in health and disease, from the microscopic elementary atom of each organ, up to his full development, and arrangement into families, tribes, and nations. Medical chemists, day and night, amid the machinery of their laboratories, hunting nature in her hidden recesses, and exposing the principles and laws of combination; medical microscopists finding beauty of form and structure, where the naked eye sees not at all, or sees only a confused speck; observing changes in texture and growth, and developing principles and systems, as wonderful in their minuteness, as that of astronomy in its magnitude. The Anatomist, the Physiologist, the Pathologist, concentrating all their powers and observations upon the various subdivisions of these extensive sciences. These men are occupied in gathering in discoveries, in trying supposed truths, with every precaution against fallacy, and then sending forth the proven results of their investigations.

The medical science of the present time, is distinguished from the practice of the earlier ages, by this fact, that it has been joined to, or proceeds from the natural sciences. What is it that has corrected our medical teaching and practice and given such impetus to our medical progress, that has revolutionized our systems and started us anew in the study of diseases, and their modes of cure, carrying away the dogmas and isms of past generations, and subjecting the present principles of medicine and surgery to the critical tests of experimental research? You are all now ready to the answer. Careful and intelligent observation of nature, has taught us all, or nearly all we know, and since we have discovered the fountain of real knowledge and have improved our instruments and modes of observation, we have truly grown much wiser, more clearly understanding what is truth, and the great care necessary to abtain it. Physiology is comparatively a new science, but it has already added to our knowledge of disease and its modes of prevention and cure, many of the most important facts, establishing principles of paramount value, and undermining false theories which had long been cherished. The study of *life in nature*, may be regarded in the practice of medicine, as the compass, rudder, and chart in navigation, showing the dangers of the sea, and guiding to the polar-star of truth. Anatomy has long been recognized as the basis of medical and surgical knowledge, and its necessity is too obvious to admit of comment. It is readily apparent how surgical practice depends upon it, and how medical knowledge also derives from it much of its accuracy and force. Chemistry also furnishes not only principles in medicine, but our means of curing disease—is at once the source of our principles and practice of medicine. Thus it is seen how legitimate medicine is founded in the natural sciences; how it stands by no assumed or supposed truths, but *is truth* itself, and much of it as capable of demonstration, as are the problems of mathematics.

In the survey of his profession, the physician sees yet other important aids and appliances. What railroads, steamships and telegraph lines have done for commerce and trade, the arts of printing, photographing and electrotyping have done for medicine. Associations are now issuing annual volumes of their transactions, giving permanent record to every established fact, and to all subjects worthy of further investigation; medical conventions and national associations, giving abiding place to every subject which has the least claim to respect, and constantly adding to, revising or correcting every principle of practice, or therapeutical conclusion, which the most careful examination shows capable of revision or improvement. In the various modern languages, we have going forth, through the channels of the medical press, and bearing to the most remote and humble member of our profession, whatever of fact, truth and wisdom has been discovered, tested, and found worthy of permanent record. Most of the medical journals of all countries are in charge of men of eminent literary and professional ability, and all of the leading ones will be found upon careful examination, to contain evidences of thought, study and investigation, as well as of intellectual ability, of which not only the profession, but human nature may well be proud. They embody the new discoveries, medical and surgical reports, bibliograghical notices of standard works. in every department of science and medicine, and are the repositories of all real progress in medicine and surgery. Standard works, illustrating with life-like exactness every form and phase of disease, describing every symptom and the effects of all known remedies, are issued in vast numbers and in all languages. Printed texts, photographed likenesses of rare and important forms of disease or injury, rendered permanent and capable of being repeated in countless numbers, thus supplying all who wish, making the combined learning, experience and wisdom of the world available to all faithful, earnest seekers after truth.

Could all see the profession of medicine in its true light, could they comprehend the magnitude of its operations, and the learning, faithfulness and fidelity with which the search for truth is made, I could this evening draw a picture of your future lives far different from what I am now compelled to foreshadow. There are not wanting those, who charge upon your profession, limited selfish, and interested motives, opposed to truth, and ready to adhere to *old* and *obsolete* opinions. Nothing saves those who utter such illiberal sentiments from contempt, but the total blindness in which they are uttered—a blindness which pertains to those not educated in the profession, and not living under its obligations. The professional man sees with an enlarged vision into regions closed to his unprofessional brother, observes the results of a vast system of intellectual machinery, while the latter forms his conclusions from a few crude and disconnected facts, or supposed facts, seen only in the limited circle of his own untutored experience and observation. It is as though one upon an eminence looks over a broad landscape, and speaks of its brilliant and varied scenery, while the man blind from birth obstinately contends that no such things exist, and that all is one uniform darkness, or as one by unaided vision sees in the pupil of the eye, a circular opening and a dark back-ground, while with the aid of our art and the use of the ophthalmoscope, you are clearly discerning the circulation in the vessels of the retina, and looking in upon the movings of one of the most intricate, delicate, and beautiful organs which the wisdom of the Almighty has ever created. The rude savage gazes at the blue arch above him, and sees only the fancied regions of future hunting-grounds lighted by dim and unknown tapers, but the astronomer with telescopic vision, sees worlds and systems of worlds, no less real than our own. While then, you are in possession of clearly-defined and well-demonstrated truth, *forgive* those who dwell in darkness and undisturbed ignorance.

There is imposed upon the science of medicine a no less boundary than pure truth; all which comes within its scope is yours, and belongs to legitimate medicine. Allow no one then to believe that you are from any cause unwilling to know what is truth, for this is a principle implanted in the human mind, which in all countries and ages, and in all sciences, has held its ground, and will at length assert its power against every opposing influence. To attain this, is directed the undivided efforts of the best, most highly educated, and most earnest men in our profession. The best minds in the world are engaged in the pursuit, stimulated by ambition, rivalry and an unselfish purpose to discover truth and banish ignorance and error. A profession, such as yours, rests upon principles far above schools, dogmas and isms. It has every protection against fallacy which human reason can know; is independent of the teaching of individual genius, and the follies and absurdities of unworthy members. It includes within its scope every principle which can be established, and every system of practice which can be shown to commend itself to an enlightened judgment. It is yours to seek for truth wherever it may be obtained; to amass knowledge from every available source; to draw wisdom from all open fountains.

It is familiar to you, how counterfeit physicians have always practiced by the side of the real, and have in all ages and nations stamped history with their impositions. In the early history of medicine and mankind, all science, morality and religion, were wrapped up in the dogmas of the schools, and traditions of the fathers, and men surrendered their minds to superstition, absurdity and vague unreasoning conjecture. Astronomy and chemistry, so exact now, wandered in the mazes of astrology and alchemy. Visionary theories agitated the profession of medicine, and as there was no law of truth and fact, by which to try them, there could be no limit to the wildness of unrestrained imagination. Now, all true science repudiates systems and theories, or only recognizes them as based upon established facts. That investigation which works within any exclusive system, is too limited and narrow to embrace the whole truth; a system can no more contain the whole science of medicine, than can the less contain the greater. Your profession rests upon certain well-determined facts, and there are various theories which have been drawn from them by different individuals, and investigation is carried on in various directions, with the view to confirm or confute these theories. Such, however, are very different from the wild schemes and visions which have been engendered, and the facts imagined or asserted to sustain them—theories and schemes which now lie scattered in ruin, monuments of warning to all who dare exclude the ground-work of truth, presuming to build permanent structures upon imaginary and vague foundations. The science of medicine, by its very nature, by the principles which govern the human mind, by every consideration of interest and ambition, can limit itself to nothing short of all attainable truth, and cannot be limited by, or bound by any system.

Such, then, is the profession you have chosen, and upon whose duties and responsibilities you now enter. As you are to meet with some embarrassments and disappointments growing out of the nature of your profession and the relations which you sustain to the public, it is but natural that I should briefly indicate their character and the causes from which they spring. You commence the practice of medicine with the sealed approval of the Faculty and other officers of the Medical department of the University of Buffalo, and it will everywhere be accepted as proof of creditable attainment in your art, but it does not confer age, experience, or that entire confidence in the public, which is the only passport to the care of those whose lives are in peril. In other words, you are yet to gain your professional standing with the public, and often to wring it as an unwilling tribute to your faithfulness and zeal, from a reluctant and unappreciating people. "It is told of a wealthy, impudent and influential quack, who, being called upon by a wondering friend who had known his former low estate and great ignorance, asked, how with so few claims and merits have you risen to fame and fortune? He took his friend to the window overlooking a crowded London street, and asked, how many wise or sensible persons may be supposed to be in the passing crowd? Not more than one in a hundred, was the reply. He says, the remainder are mine." This, though perhaps a little overdrawn, is a sad but truthful commentary upon the mental acuteness of mankind. A love of the marvelous, a blind faith in the unknown and intangible, a confidence in traditions and superstitions, and an inexplicable readiness to adopt opinions without evidence, often in opposition to the clearest and most positive proof, constitutes the basis—the stock in trade — the chief reliance in medical imposition. It would seem that after so many years of teaching by the profession, the public would understand more of disease than it does, and have more sensible and rational notions of its causes, modes of termination, and means of cure. The influence of the medical profession over the public mind, has always been controlling in everything of science or art, in all hygienic and sanitary law, in everything in which it has harmoniously and consistently exercised its power. Why is it, then, that so far in the progress of the world, we are yet so primitive and simple in this respect? Why is it that inconsistencies and absurdities which would have disgraced the earliest races of men, find believers and advocates at the present time?

I have indicated some of the causes, so far as the *public* is concerned, and in this I was intentionally very brief. There are reasons which belong to the other side—to those who have, or should have been, the educators of the public mind, and as you are hereafter to stand in this relation, it is more appropriate to dwell upon these causes of failure. It is not to be concealed, that a great many physicians have, to some extent, practiced the modes of quacks, or rather, quacks have imitated the manners of physicians who made extravagant and untruthful pretensions, which were well calculated to mislead, and have misled the people in a great degree, giving a blind faith instead of a correct understanding. But a few years since, for an intelligent patient to inquire of the nature of his disease, and especially of the proposed means of cure, was looked upon as an imposition, often construed as betraying want of confidence, and at least, as uncourteous and impolite, meriting some curt and unsatisfactory answer; such was the general manner of the medical teacher of the public. Who in the profession, faithfully and truthfully spoke of disease, telling of its origin, progress and natural termination in health? Who ever instructed the people of possible recovery without the aid of medicine, even in self-limited diseases? Who described the true nature of remedies, and the effects which might reasonably be expected to follow their use? And where, O! where, could a physician be found, who, when consulted in disease, gave *advice* instead of medicine, or colored water or bread pills? who plainly, truthfully and fearlessly explained the uselessness, in such case, of remedies, and the probable progress and termination of the disease? Bread pills and colored water in the profession, has found its counterpart and imitation out of it, in sugar pills and pure water; but who, O! who, has ever taught, or can teach the public, the immeasurable swindle of both? and when will that medical millenium appear, when those who advise the sick, will attempt no imposition upon their credulity and give a sufficient reason for their opinions? I have intimated that the prevalence of public error in medicine, is due in part, to the false reasoning and untruthful teaching of the profession, and as a natural consequence you will infer, that in so far as you would aid the prevalence of truth and banish from the public mind whatever is founded in error, you will adhere to it under all circumstances, never hesitating to risk your reputations upon its standard. For a physician to claim for himself or his art, anything not rightfully belonging thereto, is wholly inconsistent with the high obligations he is under to himself, his profession and the world.

I have thus briefly indicated what the profession is, which you have chosen, in its true dignity, strength and purity, and what its unworthy members may become, by neglect, short-sighted selfishness and down-right imposition. Nominal membership in an honorable profession, will not raise you above the level—the low level—of the charlatan and impostor, if you follow similar practices; but if you disdain to deceive, unhesitatingly adhere to the truth, both in your representations of disease, and your means of cure, reject all pretense not founded in fact, and discharge the duties of your profession intelligently, faithfully and truthfully, then I welcome you to all the honors we have this day conferred upon you, and all the rewards which gather as a halo of light around the brow of the *earnest, truthful, intelligent* physician. To become distinguished for extensive knowledge, or great wisdom—to be known widely as capable of wisely adapting means to the removal of disease, or skilfully determining its nature and cause, are objects of just and manly ambition. To obtain an enviable reputation, is the common and almost universal object of the members of your profession, much of it growing out of a natural desire to succeed in business and to gratify a personal pride and ambition. The means for accomplishing this end are carefully considered, and the surest and safest, as well as readiest and easiest, are fully estimated, often, however, forgetting the old and true maxim—"whoever would gain the rewards of diligence must suffer its fatigues." Far from intimating that the members of the medical profession are too ambitious for fame or business success. or any of the rewards of great merit, I am rather compelled, reluctantly to admit, that very many are ready to settle into a routine of thought and practice, neither benefiting themselves or growing more capable of doing good to others. I am pained to believe that some in our profession, are still using the same infallible and unvarying calomel and jalap, like the unchanging Democrat who still casts his vote for Andrew Jackson for President. Dead theories and dead practices, dead hundreds of years too late, yet long enough since to be covered in the grave of forgetfulness, thus find place and memory in the profession. To these men, recent discoveries in medicine and surgery, are new-fangled notions, unimportant and useless; improved instruments and methods of investigating disease and of making diagnosis positive, are in their estimation, only complicated machines which add nothing to the practical value of our art. Estimating the frequency of the pulse, and observing the condition of the tongue, affords them satisfactory knowledge of nearly all forms of disease, and leaves little more to be desired. The newly published books are presumed to contain little new to them, and medical journals nothing of interest. These, and such as these, have been the medical educators of the public in too great degree, until in recent years they have been gradually superceded by more active, intelligent and worthy men, and it is believed that the influence which these are exerting, will manifest itself in truer appreciation of the real value of legitimate medicine, and the total worthlessness of all isms, and pretended systems, which do not come within its sphere, and cannot be regarded as commending themselves to the approval of an enlightened understanding.

There are many ways which are constantly being tried, to obtain professional reputation and fame, while in reality there is but one avenue to its possession. Some of these various roads do lead to temporary notoriety, but never, any of them end in true professional worth, and thus, obviously, not in high honor. It is scarcely necessary to enumerate these false passages in professional life, and still, as a word of warning it may be well to briefly sketch some of them. Every young man in commencing business, must compete with other members of the profession and be compared to, and estimated in connection with them. One of the most fearful and fatal passages, thus opened up for him, and others in his interest, is detraction from the worth of his rival, and undue and exaggerated representations of personal merit. *Speak kindly of your opponents, and, above all, not boastingly of yourselves.* He is also expected in commencing business, to meet the requirements of social and religious life; the club, secret or political society and church, are at once presented, as avenues to acquaintance, social position and oftentimes professional preferment, but they are all, also, false passages and the character of their rewards well known. I do not oppose membership in these for legitimate ends, but seeking them for purposes of professional advantage, is *certain* to end in disappointment. It is true that *friends* as they patronizingly call themselves, may be gained thus, and patrons sometimes, who will "*damn you with faint praise*," and urge your preferment, because you belong to their club, lodge, or church, they will temporarily favor you because you join their circles, but they will call your rival when in imminent peril, because he belongs to the profession, and the profession to him—has no narrow, sectarian dependencies, and stands erect and firm among his fellow men, trusting in the great "Fatherhood of God" and believing in the true fellowship of men. Such alliances for professional advancement are to be avoided; reverently it may be said, that even the holy record of church influence cannot save such narrow-minded selfishness from contempt, and when proposed by the one, and accepted and acted upon by the other, shows by what dim light both are led, and by what ready means the synagogue of Christ is occupied by satan.

Physicians are to be men with other men, to join in their amusements, be interested in their welfare, to be first in benevolence, charity, education, morality, religion—to be *educators* of the people, not only in medicine, but in all respects to sustain the character and discharge the obligations which naturally devolve upon men of culture, education and attainment. They have duties to society and the world, as well as duties to themselves and their profession, and all these obliga-tions are to be discharged with faithfulness and fidelity; but *true honor* in your profession cannot spring up by accidental influence—cannot be conferred by any unnatnral or uncertain force. *True fame* grows as years and centuries grow—grows by earnest and continuous thought, by constant and patient study, by faithful and unselfish effort. With these alone it is yours. Your deed of titleship is already made out, but you must inscribe with your own hands to its conditions. You must rise up early and sit up late, you must work diligently and faithfully, and it is yours.

But I must not detain you longer from the warm greeting of friends, and the cordial welcome of all to the new field now open before you. In behalf of my colleagues and myself, I welcome you to membership in an honorable profession, and to all the high and holy trusts which are to be confided to your keeping. Impelled as I know you are, by a desire to excel in your profession, looking upon it, not as a trade, but as a high mission, and desirous of adding to its intelligence and worth, rather than of receiving from it undeserved favor, I bid you a most hearty welcome—welcome to all the honors and rewards which spring from high aims, and pure purposes—to all the pleasures and joys which flow from the nearest fellows of friendship, the kindest sympathies and interests of those who receive your ministrations, and welcome to a faithful keeping of their most sacred trusts; for into your hands will be committed interests dearer than life. I must also bid you welcome to all the toils and hardships, to all the watchings and anxieties, all the trials and disappointments, all the labors und dangers of professional life. Dangers, did I say? Yes, dangers. Your profession has its martyrs, a great company whose names are written so high as never to be forgotten. They were inoculated with disgusting and fatal diseases, pursued their calling side by side with the pestilence, until the fearful struggle ended in death, or lost their lives in defense of their country. Their heroic self-sacrifices embalm their memories, and are more enduring monuments than bronze or marble. Again, thrice welcome to the companionship of those you most admire and honor; to a share in like labors and rewards—delving in the same mines of truth, and drinking from the same fountains of knowledge.

Wherever Providence assigns your lot, accept its conditions, and cheerfully, hopefully and earnestly pursue your calling. In prosperity remember that the jealous eye of your *Alma Mata* will steadily watch your progress, and claim your successes as her own—will take pride and pleasure in whatever promotes your real prosperity. In times of difficulty and trial, remember, also, that a cordial hand of good fellowship will be extended, to defend you when right, and shield and correct you if wrong. Go forth, then, on your mission of love and good will to men, neither dismayed by temporary and apparent defeat, or over elated by unexpected success.

> "With wing on the wind, and eye on the sun,
> Swerve not a hair but press onward, right on."

And—

> "In the tempest of life, when the wave and the gale
> Are around and above, if thy footing should fail,
> If thine eye should grow dim and thy caution depart,
> 'Look aloft,' and be firm and be fearless of heart."

---

THE "International Societies for aid to the Wounded in Time of War" have awarded to Mr. Condy, of Battersea, their medal, in recognition of the importance to military surgery of his discovery of the disinfecting properties of the alkaline permanganates, and the great sanitary value of Condy's fluid, as proved by the experience of the Prussian army surgeons during the late Bohemian war.

